# *Ficus carica* Latex Modulates Immunity-Linked Gene Expression in Human Papillomavirus Positive Cervical Cancer Cell Lines: Evidence from RNA Seq Transcriptome Analysis

**DOI:** 10.3390/ijms241713646

**Published:** 2023-09-04

**Authors:** Muharrem Okan Cakir, Ugur Bilge, Declan Naughton, G. Hossein Ashrafi

**Affiliations:** 1 School of Life Sciences, Pharmacy and Chemistry, Kingston University London, London KT1 2EE, UK; m.okan@kingston.ac.uk (M.O.C.); d.naughton@kingston.ac.uk (D.N.); 2 Department of Biostatistic and Medical Informatics, Faculty of Medicine, Akdeniz University, 07058 Antalya, Turkey; ubilge@akdeniz.edu.tr

**Keywords:** cervical cancer, fig latex, *Ficus carica*, RNA-seq, pathway enrichment, high risk HPV, antigen presentation, antigen processing

## Abstract

Cervical carcinogenesis is the leading cause of cancer-related deaths in women, and the role of high-risk human papillomavirus (HR-HPV) as a possible risk factor in the development of this cancer is well recognized. Despite the availability of multi-therapeutic approaches, there is still major concern regarding the prevention of metastatic dissemination and excessive tissue injuries. Therefore, it is imperative to develop a safer and more efficient treatment modality. *Ficus carica*, a natural plant, has shown potential therapeutic properties through its fruit latex when applied to HPV-positive cervical cancer cell lines. However, the mechanisms of action of *Ficus carica* (fig) latex are not well understood. This study aims to provide a deeper insight into the biological activities of fig latex on human cervical cancer cell lines expressing high-risk HPV types 16 and 18. The data obtained from this study reveal that fig latex influences the expression of genes involved in “Class I MHC-mediated antigen presentation” as well as “Antigen processing: Ubiquitination and Proteasome degradation”. These genes play a crucial role in host immune surveillance and the resolution of infection. Notably, Western blot analysis corroborated these findings, demonstrating an increase in the expression of MHC class I in HeLa cells after fig latex treatment. Findings from this study suggest that fig latex may enhance T cell responses against oncogenic HPV, which could be beneficial for the clearance of early-stage cancer.

## 1. Introduction

Cervical cancer is one of the most common cancers affecting women worldwide, with an estimated 604,127 new cases and 341,831 deaths in 2020 alone [1]. It is primarily caused by infection with high-risk human papillomaviruses (HPVs), which can lead to persistent lesions and the progression of cervical cancer [2,3]. Insufficient immune clearance of HPV-infected cells prevents the effective elimination of the virus, contributing to the development and progression of cervical cancer [3]. One of the key factors responsible for this immune clearance failure is the E5 protein of HPV. We have previously shown that E5 is expressed during the early stages of infection and downregulates the transport of MHC class 1 complexes to the cell surface [4,5]. The downregulation of cell surface MHC class I may allow the virus to establish infection by avoiding immune clearance of virus-infected cells via cytotoxic T cells (CTLs) [6]. Therapeutic attempts have been made to treat malignant diseases caused by HPV through vaccines/immunotherapy. However, it has met with limited success due to local and systemic immunosuppressive factors in HPV-positive tumors [7,8,9]. Therefore, there is a growing demand for novel and safer therapeutic approaches against HPV-related cervical cancer [10,11].

In recent years, natural products derived from plants have gained considerable attention for their potential therapeutic properties against various types of cancer, including cervical cancer [12,13]. Many natural compounds have been identified as potential candidates for cancer therapy due to their ability to induce apoptosis, inhibit cell proliferation, and modulate cellular signaling pathways [14]. Among these, fig latex derived from *Ficus carica* has exhibited promising anticancer properties. Recent studies have highlighted the cytotoxic effects of fig latex against various cancer cell lines, including cervical, stomach, and colorectal cancer [15,16,17]. Notably, our previous publication demonstrated the effectiveness of fig latex in suppressing cervical cancer cell growth and inducing apoptosis, thereby substantiating its potential as a valuable candidate for cancer therapy [17].

Based on the above highlights, we investigated the effects of fig latex on immune response-related genes in HPV-related cervical cancer by using RNA sequencing (RNA-seq). Here, we show that fig latex effectively inhibits the growth of HPV-positive cervical cancer cell lines, namely CaSki and HeLa, while having no cytotoxic effects on normal/non-cancerous cervical cells (HCKT1). Analysis of RNA-seq data revealed that fig latex regulates the expression of genes associated with immune surveillance, specifically those involved in “Class I MHC-mediated antigen presentation” as well as “Antigen processing: Ubiquitination and Proteasome degradation”. These findings shed light on new therapeutic avenues against HPV-associated cervical cancers. The outcomes of this study hold promise for the expedited resolution of HPV infection, particularly benefiting individuals with early-stage HPV-related cancer.

## 2. Results

### 2.1. Fig Latex Inhibits the Growth of Human Cervical Cancer Cell Lines

**Figure 1:**
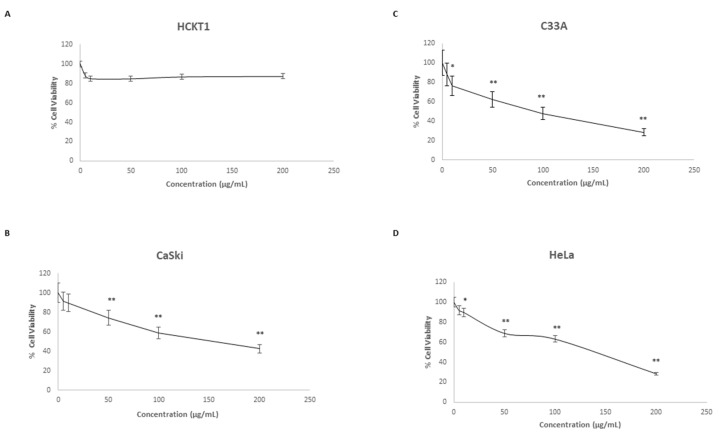
The effect of fig latex on the growth of cervical cell lines. (**A**) HCKT1, (**B**) CaSki, (**C**) C33A, and (**D**) HeLa were treated with different concentrations of fig latex (5 μg/mL, 10 μg/mL, 50 μg/mL, 100 μg/mL, and 200 μg/mL) for 72 h. The SRB assay was used to determine cell viability. Data points represent the mean ± SD of three independent experiments. IC50 values were calculated by R computing software using a sigmoidal curve fit based on nonlinear regression. Statistical significance was assessed via a one-way ANOVA test followed by a Tukey post hoc test and represented as follows: * *p*  <  0.05 and ** *p*  <  0.01 vs. fig latex 0 μg/mL in DMSO.

To investigate whether fig latex affects the growth of four distinct cervical cell lines, one normal human cervical keratinocyte (HCKT1), two HPV-positive cervical cancer cell lines (HeLa HPV18+, CaSki HPV16+), and one HPV-negative cervical cancer cell line (C33A) were treated with various concentrations of fig latex (5 μg/mL, 10 μg/mL, 50 μg/mL, 100 μg/mL, 200 μg/mL) for 72 h. The viability of the cells was analyzed using a Sulforhodamine B (SRB) colorimetric assay. The results revealed that the IC50 values of fig latex at 72 h on HeLa, CaSki, and C33A were found to be 106 μg/mL, 110 μg/mL, and 108 μg/mL, respectively (Figure 1B–D). Moreover, fig latex did not induce cytotoxicity in normal human cervical keratinocytes compared to cervical cancer cells (Figure 1A). These findings suggest that certain concentrations of fig latex exhibit selective cytotoxicity towards HPV-positive cervical cancer cells while sparing normal cells.

### 2.2. Transcriptomic Profiling of Different HPV-Positive Cervical Cancer Cells upon Fig Latex Treatment

To study the gene expression profile of HPV-positive human cervical cancer cells under fig latex treatment, cervical cancer cell lines, HPV-18 type positive HeLa, HPV-16 type positive CaSki, and HPV-negative C33A were treated with 100 μg/mL of fig latex that was closest to the IC50 value of fig latex. The gene expression analysis was performed using RNA-seq.

**Figure 2:**
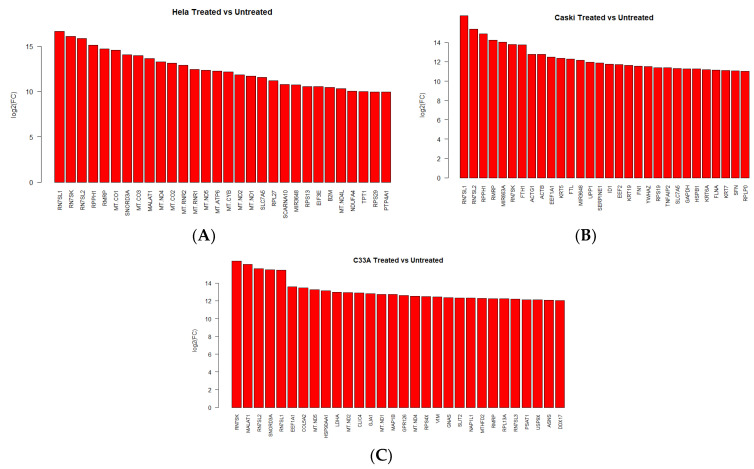
Gene expression signature differences in (**A**) HeLa, (**B**) CaSki, and (**C**) C33A cell lines using the 30 most upregulated genes after 72 h of fig latex treatment. The expression of individual genes (log2FC) across both cell lines is shown in red.

Upon fig latex treatment, we examined the individual gene expression patterns in the HeLa, CaSki, and C33A cell lines. In HeLa cells, we observed a significant increase in the expression levels of genes RNSL1, RN7SK, and RNS7L2 in response to fig latex treatment. Similarly, in CaSki cells, we identified a distinct set of upregulated genes, including RNS7L1, RNS7SL2, RPPH1, and RMRP (Figure 2A,B). On the other hand, in C33A cell lines, several genes, including RN7SK, MALAT1, and SNORD3A, were found to be upregulated (Figure 2C).

Furthermore, functional enrichment analysis was conducted separately for upregulated genes in the Hela, CaSki, and C33A cell lines. In Hela cells, the analysis revealed significant enrichment of genes associated with the ribonucleoprotein complex, signal recognition particle, and RNA processing among the top functional categories. Similarly, in CaSki cells, functional enrichment analysis showed significant enrichment in genes related to the ribonucleoprotein complex, the antimicrobial immune response mediated by antimicrobial peptides, and the signal recognition particle (Table 1, parts A and B). Conversely, the C33A cell line, which lacks HPV infection, exhibited changes in pathways related to nonsense-mediated decay, apoptosis, and the unfolded protein response (Table 1, part C).

These findings suggest that the effects of fig latex on immune-related genes are HPV-specific rather than exclusive to cancer-related alterations.

### 2.3. Analysis of Differentially Expressed Genes in HPV-Positive Cervical Cancer Cell Lines upon Fig Latex Treatment Using Pathway Enrichment Analysis

Differential gene expression analysis showed 149 significantly differentially expressed genes, which were expressed in HPV-positive cancer cell lines. In total, 64 differentially expressed genes were consistently downregulated, while 85 of them were consistently upregulated in HPV-positive cervical cancer cell lines upon fig latex treatment (Supplementary Table S1).

Differentially expressed genes in HPV-positive cervical cancer cell lines following fig latex treatment were analyzed using pathway enrichment analysis. The results demonstrated that the genes regulated by fig latex treatment were prominently involved in immune surveillance pathways, including “Class I MHC-mediated antigen presentation” and “processing through ubiquitination and proteasome degradation” (Table 2). Importantly, we observed a set of common genes that overlapped across these pathways, suggesting their pivotal role in viral immune response. These common genes included *RPS27A*, *RNF111*, *CUL5*, *FBXO4*, *KLHL22*, *FBXL4*, and *TRIP12*. The findings from this pathway enrichment analysis suggest that fig latex may modulate key signaling pathways involved in viral immune response, highlighting its therapeutic potential in the management of HPV-associated cervical cancers.

### 2.4. Analysis of MHC Class I Expression in HPV-Positive Cervical Cell Lines upon Fig Latex Treatment

**Figure 3:**
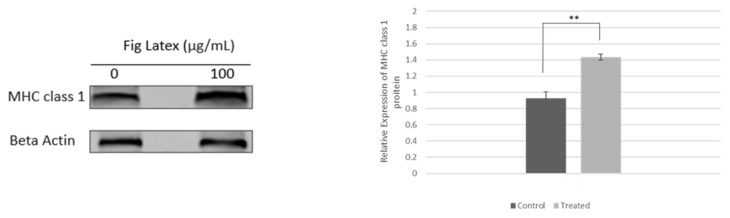
Effect of fig latex on the expression of MHC class 1 in HPV-positive HeLa cells. The cells were treated with 0 μg/mL (control) and 100 μg/mL of fig latex for 72 h. The expression of MHC class 1 was analyzed via Western blot. The expression of beta-actin was used as a loading control. Western blotting results are representative of the results obtained in three separate experiments. Densitometry indicating relative band expression for each blot measured using ImageJ software (version number: 1.54f). Statistical significance was assessed via Student’s *t*-test and represented as follows: ** *p*  <  0.01 vs. fig latex 0 μg/mL in DMSO.

The results of RNA-seq revealed that fig latex induces the expression of genes related to MHC class 1-mediated antigen presentation and processing. We examined the expression of MHC class 1 in HPV-positive HeLa cells after fig latex treatment. As shown in Figure 3, fig latex induced the expression of MHC class 1 in HeLa cells. This finding suggests that fig latex may counteract the effect of HPV E5 protein and restore the expression of MHC class 1-mediated antigen processing and presentation.

## 3. Discussion

Cervical cancer, primarily caused by high-risk human papillomavirus (HPV) infection, represents a significant global burden [1,18]. A major challenge in the treatment of cervical cancer lies in the tumor cells’ ability to evade immune surveillance, mainly mediated by HPV oncoproteins E5 [4,5,6,7]. This immune evasion not only allows the tumors to evade destruction by the immune system but also contributes to the progression of the disease, leading to poor clinical outcomes [3,7]. Consequently, the development of novel therapeutic interventions is crucial [18]. Natural products have gained considerable attention in cancer research due to their potential as sources of novel therapeutic agents [14]. Our previous study demonstrated the potential therapeutic properties of *Ficus carica* latex, a natural product derived from fig, when applied to HPV-positive cervical cancer cell lines [17]. However, the specific molecular mechanisms underlying its action in HPV-positive cervical cancer cells remain largely unknown. In this study, we aimed to investigate the potential of fig latex as a therapeutic intervention to counteract immune evasion and enhance the immune response against HPV-positive cancer cells.

To achieve this, we subjected HPV-positive cervical cancer cells to fig latex treatment and examined the gene expression profile and signaling pathways involved in immune surveillance. Through comprehensive RNA-seq analysis, we thoroughly examined the transcriptome of the treated cells and compared it to the control group. This approach allowed us to identify differentially expressed genes and gain insights into the molecular changes induced by fig latex treatment.

Our study demonstrated the growth-inhibitory effects of fig latex on cervical cancer cells, which aligns with our previous findings [17]. Treatment with fig latex significantly inhibited cell growth in HeLa, CaSki, and C33A cells, with calculated IC50 values of 106, 110, and 108 μg/mL, respectively. Importantly, normal human cervical keratinocytes (HCKT1) were unaffected by fig latex treatment, indicating its selective cytotoxicity towards cervical cancer cells. These findings suggest that fig latex possesses potent anti-cancer properties, specifically targeting cervical cancer cells while sparing normal/non-cancerous cells.

In addition to its growth inhibitory effect, this study reports, for the first time, that fig latex exerts anticancer effects by modulating key signaling pathways involved in immune surveillance, including the Class I MHC-mediated antigen processing and presentation pathway (*p*-value: 7.82 × 10^−8^) and the antigen processing ubiquitination and proteasome degradation pathway (*p*-value: 1.12 × 10^−6^). The Class I MHC-mediated antigen processing pathway is crucial for immune recognition by presenting antigens to T cells. Notably, several key genes involved in antigen presentation, including *RPS27A*, *RNF111*, *CUL5*, *FBXO4*, *FBXL4*, and *CALR*, were upregulated upon fig latex treatment [19,20,21,22,23,24]. It is well documented that HPV E5 protein hinders major histocompatibility complex (MHC) class 1 antigen presentation by impeding the transport of MHC class 1 molecules to the cell surface, thereby compromising the recognition of HPV-infected cells by cytotoxic T lymphocytes (CTLs) and dampening the antiviral immune response [4,5,6]. The restoration of antigen presentation by fig latex treatment in HPV-positive cervical cancer cells signifies its ability to overcome the inhibitory effects of HPV E5 and reinstate immune recognition of infected cells. By modulating the Class I MHC-mediated antigen processing and presentation pathway, fig latex treatment potentially enhances immune recognition and promotes the clearance of HPV-infected cells. This is consistent with other studies indicating that restoration of antigen presentation renders virus-infected cells more susceptible to host immune responses [25,26]. Moreover, the antigen processing (ubiquitination and proteasome degradation) pathway (*p*-value: 1.12 × 10^−6^) was also significantly enriched. This pathway plays a critical role in antigen processing and presentation, influencing immune recognition and response. Several key genes involved in ubiquitination and proteasome degradation were found to be upregulated, including *RPS27A*, *RNF111*, *CUL5*, *FBXO4*, and *FBXL4*. These genes contribute to the regulation of protein degradation, ensuring the proper turnover of antigens for presentation by MHC molecules [27,28]. In the context of HPV and cancer, HPV infection can dysregulate antigen processing and presentation, allowing infected cells to evade immune recognition [29,30]. The identification of upregulated genes within the antigen processing ubiquitination and proteasome degradation pathway suggests that fig latex treatment may counteract the HPV-induced disruption of antigen processing and presentation. Of note, fig latex treatment did not induce immunity-related gene expression in C33A cells, which lack HPV infection. This highlights fig latex’s impact on immune-related genes. The findings from the analysis of common genes in HPV-positive cancer cell lines upon fig latex treatment align with the individual gene expression patterns observed in HeLa and CaSki cells following fig latex treatment. Collectively, the consistent upregulation of immune-related genes and genes involved in ribonucleoprotein complexes and RNA processing across both individual and common gene expression patterns emphasizes a potential enhancement of the immune response against HPV infections, which is linked to HPV presence rather than being exclusive to cancer-related alterations.

In particular, our RNA-seq results initially revealed the upregulation of genes associated with MHC class I-mediated antigen presentation and processing following fig latex treatment in HPV-positive cervical cancer cell lines. Moreover, our Western blot analysis corroborated these findings by demonstrating a notable increase in the expression of MHC class I in HeLa cells subsequent to fig latex treatment. This parallel validation through Western blot analysis further bolsters the robustness of our results and underscores fig latex’s potential as an immunomodulatory agent in the context of HPV-positive cervical cancer cells.

In conclusion, our findings provide valuable insights into the molecular mechanisms underlying fig latex’s action and its immunomodulatory potential in combating HPV-positive cervical cancer. By targeting immune evasion mechanisms and enhancing immune recognition, fig latex or its active components hold promise for the development of novel therapeutic strategies against cervical cancer.

## 4. Materials and Methods

### 4.1. Chemicals and Reagents

Cell culture medium, Dulbecco’s modified Eagle medium (DMEM), Roswell Park Memorial institute (RPMI) 1640 medium, keratinocyte serum-free medium (SFM), and supplements including EGF (epidermal growth factor) and bovine pituitary extract (BPE), penicillin-streptomycin, trypsin, Dulbecco’s phosphate-buffered saline (DPBS), and Sodium pyruvate were purchased from Gibco (ThermoFisher, Oxford, UK). Y-27632, a Rho kinase inhibitor, and a Sulforhodamine B (SRB) assay kit were purchased from Abcam, Cambridge, UK. Dimethyl sulfoxide (DMSO) and fetal bovine serum (FBS) were purchased from Sigma, Welwyn Garden City, UK. A GenElute RNA/DNA/Protein Purification Plus kit was purchased from Sigma-Aldrich, UK. M. Anti-MHC class 1 antibody and anti-actin antibody were purchased from Abcam, UK. Bovine serum albumin (BSA) and TBS-T were purchased from Thermofisher, UK.

### 4.2. Collection and Purification of Ficus carica Latex

*Ficus carica* latex was collected drop by drop without squeezing over the summer months from the unripe fruits of fig trees in the suburb of Antalya, Turkey. We performed the purification of fig latex as described in our previous study [17]. Briefly, the latex was initially filtered using a Whatman No. 1 filter from ThermoFisher Scientific, UK. After filtration, it was then centrifuged at 13,000 rpm at a temperature of 4 °C to separate the polymeric gum from the liquid filtrate. The aqueous part was further purified via filtration using a disposable filter membrane with a pore size of 5 µm from Sigma, UK. It was stored at −20 °C for further analysis.

### 4.3. Cell Lines and Cell Culture Conditions

Human cervical cancer cell lines, namely HPV type 16 positive CaSki, HPV type 18 positive HeLa, and HPV negative C33A, were obtained from the American Type Culture Collection (Manassas, VA, USA). HPV-free human cervical keratinocytes, HCKT1, were kindly gifted by Prof. Tohru Kiyono, Japan National Cancer Center. HeLa, CaSki, and C33A cells were maintained in DMEM supplemented with 10% heat-inactivated FBS and 100 μg/mL of penicillin-streptomycin. HCKT1 cells, on the other hand, were cultured in a serum-free medium supplemented with 20 μg/mL BPE, 0.2 ng/mL, and 10 μM Y-27632. All cell lines were grown in a humidified atmosphere with 5% CO_2_ at 37 °C.

### 4.4. SRB Cell Viability Assay

In order to investigate the effect of fig latex on cell growth, a Sulforodamine B (SRB) assay was performed. For cell viability analysis, the aqueous part of the plant extract was subjected to freeze-drying to obtain a powder form. The freeze-dried powder was then dissolved in DMSO to prepare a 1 mg/mL stock solution. Several concentrations were prepared by diluting the stock solution with a cell culture medium. Human cervical cancer cells (HeLa and CaSki) and normal HCKT1 cells were cultured at a concentration of 5 × 10^4^ in 0.1 mL of medium in a 96-well plate. The following day, cells were treated with various concentrations (5, 10, 50, 100, and 200) of fig latex. After 72 h of treatment, cells were fixed with a fixation solution for 1 h. After 3 washes with distilled water, cells were stained with SRB solution for 15 min and rinsed with washing solution 3 times. Protein-bound dye was solubilized, and the optical density was determined at 545 based on the manufacturer’s recommendations. For all the experiments, the percentage of cytotoxicity was calculated as [(O.D. vehicle) × (O.D. sample)/O.D. vehicle] × 100. Background correction was carried out by subtracting the O.D. of culture media. The percent of proliferation in each treated cell line was normalized based on their control wells. All experiments were performed at least in triplicate. All treatments were adjusted to equal concentrations of DMSO between 0.1~0.2%.

### 4.5. RNA Preparation

Total RNA extraction from fig latex-treated and untreated cell lines was performed using the Gen Elute kit according to the manufacturer’s instructions. The quality of total RNA was assessed using the Agilent 2100 bioanalyzer (Agilent, Palo Alto, CA, USA) with the RNA 6000 Nano LabChip kit (Fisher Scientific, UK). All RNA samples selected for sequencing had an RIN value greater than 7.5.

### 4.6. RNA Sequencing (RNA Seq)

RNA samples were sent to CeGaT GmbH, Tübingen, Germany, for library preparation, sequencing, and bioinformatic analysis. Libraries were prepared using the SMART-Seq Stranded kit (Takara, Kusatsu, Japan). Multiplexed libraries were sequenced on the Illumina NovaSeq 6000 platform at 100 bp paired-end reads. The sequencing depth for each sample was >20 million reads. All samples passed quality control based on the manufacturer’s standards.

### 4.7. Bioinformatic Analysis

The sequence reads were analyzed further using diverse bioinformatic tools. Demultiplexing of the sequencing reads was performed with Illumina bcl2fastq (vs. 2.20). Adapters were trimmed with Skewer (vs. 0.2.2) [31]. Trimmed raw reads were aligned to hg19-cegat using STAR (version 2.7.3) [32]. Pseudoautosomal regions (PAR) were masked on chromosome Y (chrY:10001-2649520, chrY:59034050-59363566). Reads originating from these regions can be found at their respective locations on chromosome X. Normalized counts have been calculated with DESeq2 (version 1.24.0) in R (version 3.6.1) [33]. DESeq2 uses a negative binomial generalized linear model to test for differential expression based on gene counts.

For functional enrichment analysis, the RNA sequencing (RNA-Seq) data obtained from drug-treated and untreated cells were used. Gene Set Enrichment Analysis (GSEA) was performed using the GSEA software (version number: 4.3.0) [34,35]. The RNA-Seq data sets were preprocessed and normalized, and the resulting gene expression profiles were analyzed against a comprehensive collection of gene sets derived from public databases, such as MSigDB [34]. The GSEA algorithm computed an enrichment score for each gene set, indicating the extent to which the gene set was overrepresented among the differentially expressed genes.

Moreover, EnrichR, an online platform for comprehensive gene set enrichment analysis, was utilized [36,37,38]. The preprocessed RNA-Seq data sets were uploaded to EnrichR, and the analysis was conducted by following the provided instructions. EnrichR integrates multiple pathway and gene set databases, such as KEGG and Reactome, to identify enriched pathways associated with differentially expressed genes. The analysis generated enriched pathway results with corresponding statistical significance. The results obtained from both GSEA and EnrichR (version number: 3.2) were used to gain insights into the biological processes and pathways affected by the drug treatment in the cells [34,35,36,37,38].

### 4.8. Western Blotting

To investigate the effect of fig latex on the expression of MHC class 1 protein, a standard semi-dry Western blotting technique was used. HeLa cells were treated with 0 μg/mL and 100 μg/mL of fig latex for 72 h then total protein was extracted using a Gen Elute Protein Purification kit and equal amounts of proteins were electrophoresed. The membrane was blocked with 5% bovine serum albumin (BSA) in TBS–T (10 mM Tris-HCI, pH 7.5, 150 mM NaCI, and 1% (*v*/*v*) Tween 20) at room temperature for 2 h to reduce nonspecific binding and incubated with mAb anti-MHC class 1 antibody (1:500), followed by Donkey anti-mouse (1:10,000) and actin antibody (1:10,000). The membrane was then visualized using the OdysseyClx Imaging System (Li-COR).

### 4.9. Statistical Analysis

The data were collected from at least three independent experiments and presented as the mean  ±  standard deviation for each group. Statistical analyses, including one-way analysis of variance (ANOVA) followed by post hoc Tukey’s test, were conducted using R Studio software with the ‘stats’ package for ANOVA and the ‘agricolae’ package for post hoc testing. A significance level of *p* < 0.05 was considered to indicate a statistically significant difference.

## Figures and Tables

**Table 1:** The results of functional enrichment analysis for upregulated genes in HeLa, CaSki, and C33A in parts A, B and C respectively. cells after fig latex treatment. The table includes only statistically significant pathways (*p* < 0.05), presenting the top 3 enriched cellular processes, their corresponding *p*-values, and the list of overlapping genes.

**(A)**			
**Cellular Processes**	**Number of Overlap Genes**	***p*** **-Value**	**Overlap Genes**
Ribonucleoprotein Complex	9	1.45 × 10^−10^	*RPL27*, *RPS29*, *RPS13*, *EIF3E*, *RN7SL2*, *RN7SL1*, *RMRP*, *RPPH1*, *RN7SK*
Signal Recognition Particle	2	1.72 × 10^−5^	*RN7SL2*, *RN7SL1*
RNA processing	6	1.37 × 10^−5^	*RPL27*, *RPS13*, *RMRP*, *RPPH1*, *SCARNA10*, *SNORD3A*
**(B)**			
**Cellular Processes**	**Number of Overlap Genes**	***p*** **-Value**	**Overlap Genes**
Ribonucleoprotein Complex	9	4.47 × 10^−8^	*ACTB*, *RPS19*, *EEF2*, *GAPDH*, *RN7SK*, *RN7SL2*, *RN7SL1*, *RMRP*, *RPPH1*
Antimicrobial immune response mediated by antimicrobial peptides	3	2.44 × 10^−5^	*KRT6A*, *RPS19*, *GAPDH*
Signal Recognition Particle	2	4.07 × 10^−5^	*RNS7SL1*, *RN7SL2*
**(C)**			
**Cellular Processes**	**Number of Overlap Genes**	***p*** **-Value**	**Overlap Genes**
Nonsense-mediated decay	7	8.51 × 10^−9^	*RPL11*, *RPS11*, *RPL13A*, *RPS3A*, *RPS4X*, *RPS18*, *RPL15*
Apoptosis	10	6.23 × 10^−6^	*DNAJA1*, *CCND2*, *ANXA1*, *CCND1*, *CAV1*, *LMNA*, *HMGB2*, *SQSTM1*, *SPTAN1*, *CD44*
Unfolded Protein Response	8	2.09 × 10^−5^	*HSPA9*, *SLC7A5*, *XPOT*, *MTHFD2*, *PSAT1*, *ASNS*, *ATF4*, *EIF4G1*

**Table 2:** Pathway enrichment analysis of common genes in HPV-positive cervical cancer cell lines after fig latex treatment. The table includes a description of the biological pathway or process, the number of overlap genes from differentially expressed genes, the *p*-value, the FDR q-value, and the specific genes that overlap for each pathway or process.

**Description**	**Number of Overlap Genes**	***p*** **-Value**	**FDR q-Value**	**Overlap Genes**
Class I MHC-mediated antigen processing and presentation	11	7.82 × 10^−8^	2.21 × 10^−4^	*RPS27A*, *RNF111*, *CUL5*, *FBXO4*, *KLHL22*, *FBXL4*, *TRIP12*, *RNF6*, *RNF115*, *CALR*, *CTSV*
Antigen processing: ubiquitination and proteasome degradation	9	1.12 × 10^−6^	8.56 × 10^−4^	*RPS27A*, *RNF111*, *CUL5*, *FBXO4*, *KLHL22*, *FBXL4*, *TRIP12*, *RNF6*, *RNF115*

## Data Availability

The data presented in this study are available on request from the corresponding author. The data is not publicly available due to confidentiality.

## Supplementary Materials

The following supporting information can be downloaded at: https://www.mdpi.com/article/10.3390/ijms241713646/s1.

## References

[1] Sung H., Ferlay J., Siegel R.L., Laversanne M., Soerjomataram I., Jemal A., Bray F. (2021). Global Cancer Statistics 2020: GLOBOCAN Estimates of Incidence and Mortality Worldwide for 36 Cancers in 185 Countries. CA Cancer J. Clin..

[2] zur Hausen H. (2002). Papillomaviruses and Cancer: From Basic Studies to Clinical Application. Nat. Rev. Cancer.

[3] Senba M., Mori N. (2012). Mechanisms of Virus Immune Evasion Lead to Development from Chronic Inflammation to Cancer Formation Associated with Human Papillomavirus Infection. Oncol. Rev..

[4] Ashrafi G.H., Haghshenas M.R., Marchetti B., O’Brien P.M., Campo M.S. (2005). E5 Protein of Human Papillomavirus Type 16 Selectively Downregulates Surface HLA Class I. Int. J. Cancer.

[5] Ashrafi G.H., Haghshenas M., Marchetti B., Campo M.S. (2006). E5 Protein of Human Papillomavirus 16 Downregulates HLA Class I and Interacts with the Heavy Chain via Its First Hydrophobic Domain. Int. J. Cancer.

[6] Campo M.S., Graham S.V., Cortese M.S., Ashrafi G.H., Araibi E.H., Dornan E.S., Miners K., Nunes C., Man S. (2010). HPV-16 E5 down-Regulates Expression of Surface HLA Class I and Reduces Recognition by CD8 T Cells. Virology.

[7] Smola S., Trimble C., Stern P.L. (2017). Human Papillomavirus-Driven Immune Deviation: Challenge and Novel Opportunity for Immunotherapy. Ther. Adv. Vaccines.

[8] Trimble C.L., Morrow M.P., Kraynyak K.A., Shen X., Dallas M., Yan J., Edwards L., Parker R.L., Denny L., Giffear M. (2015). Safety, Efficacy, and Immunogenicity of VGX-3100, a Therapeutic Synthetic DNA Vaccine Targeting Human Papillomavirus 16 and 18 E6 and E7 Proteins for Cervical Intraepithelial Neoplasia 2/3: A Randomised, Double-Blind, Placebo-Controlled Phase 2b Trial. Lancet.

[9] Maldonado L., Teague J.E., Morrow M.P., Jotova I., Wu T.C., Wang C., Desmarais C., Boyer J.D., Tycko B., Robins H.S. (2014). Intramuscular Therapeutic Vaccination Targeting HPV16 Induces T Cell Responses That Localize in Mucosal Lesions. Sci. Transl. Med..

[10] Davies-Oliveira J.C., Smith M.A., Grover S., Canfell K., Crosbie E.J. (2021). Eliminating Cervical Cancer: Progress and Challenges for High-Income Countries. Clin. Oncol..

[11] George I.A., Chauhan R., Dhawale R.E., Iyer R., Limaye S., Sankaranarayanan R., Venkataramanan R., Kumar P. (2022). Insights into Therapy Resistance in Cervical Cancer. Adv. Cancer Biol. Metastasis.

[12] Newman D.J., Cragg G.M. (2007). Natural Products as Sources of New Drugs over the Last 25 Years. J. Nat. Prod..

[13] Cragg G.M., Grothaus P.G., Newman D.J. (2014). New Horizons for Old Drugs and Drug Leads. J. Nat. Prod..

[14] Kubczak M., Szustka A., Rogalińska M. (2021). Molecular Targets of Natural Compounds with Anti-Cancer Properties. Int. J. Mol. Sci..

[15] Soltana H., Pinon A., Limami Y., Zaid Y., Khalki L., Zaid N., Salah D., Sabitaliyevich U.Y., Simon A., Liagre B. (2019). Antitumoral Activity of *Ficus carica* L. on Colorectal Cancer Cell Lines. Cell. Mol. Biol..

[16] Hashemi S.A., Abediankenari S., Ghasemi M., Azadbakht M., Yousefzadeh Y., Dehpour A.A. (2011). The Effect of Fig Tree Latex (*Ficus carica*) on Stomach Cancer Line. Iran. Red Crescent Med. J..

[17] Ghanbari A., Le Gresley A., Naughton D., Kuhnert N., Sirbu D., Ashrafi G.H. (2019). Biological Activities of Ficus Carica Latex for Potential Therapeutics in Human Papillomavirus (HPV) Related Cervical Cancers. Sci. Rep..

[18] Burmeister C.A., Khan S.F., Schäfer G., Mbatani N., Adams T., Moodley J., Prince S. (2022). Cervical Cancer Therapies: Current Challenges and Future Perspectives. Tumour Virus Res..

[19] Antoniou A.N., Powis S.J., Elliott T. (2003). Assembly and Export of MHC Class I Peptide Ligands. Curr. Opin. Immunol..

[20] Elliott T., Neefjes J. (2006). The Complex Route to MHC Class I-Peptide Complexes. Cell.

[21] Purcell A.W., Elliott T. (2008). Molecular Machinations of the MHC-I Peptide Loading Complex. Curr. Opin. Immunol..

[22] Wei J., Kishton R.J., Angel M., Conn C.S., Dalla-Venezia N., Marcel V., Vincent A., Catez F., Ferré S., Ayadi L. (2019). Ribosomal Proteins Regulate MHC Class I Peptide Generation for Immunosurveillance. Mol. Cell.

[23] Wearsch P.A., Cresswell P. (2008). The Quality Control of MHC Class I Peptide Loading. Curr. Opin. Cell Biol..

[24] York I.A., Rock K.L. (1996). Antigen Processing and Presentation by the Class I Major Histocompatibility Complex. Annu. Rev. Immunol..

[25] Davis D.A., Shrestha P., Aisabor A.I., Stream A., Galli V., Pise-Masison C.A., Tagawa T., Ziegelbauer J.M., Franchini G., Yarchoan R. (2019). Pomalidomide Increases Immune Surface Marker Expression and Immune Recognition of Oncovirus-Infected Cells. Oncoimmunology.

[26] Davis D.A., Mishra S., Anagho H.A., Aisabor A.I., Shrestha P., Wang V., Takamatsu Y., Maeda K., Mitsuya H., Zeldis J.B. (2017). Restoration of Immune Surface Molecules in Kaposi Sarcoma-Associated Herpes Virus Infected Cells by Lenalidomide and Pomalidomide. Oncotarget.

[27] van Endert P. (2011). Post-Proteasomal and Proteasome-Independent Generation of MHC Class I Ligands. Cell. Mol. Life Sci..

[28] Hearn A., York I.A., Bishop C., Rock K.L. (2010). Characterizing the Specificity and Cooperation of Aminopeptidases in the Cytosol and Endoplasmic Reticulum during MHC Class I Antigen Presentation. J. Immunol..

[29] Đukić A., Lulić L., Thomas M., Skelin J., Bennett Saidu N.E., Grce M., Banks L., Tomaić V. (2020). HPV Oncoproteins and the Ubiquitin Proteasome System: A Signature of Malignancy?. Pathogens.

[30] Wang Y., Xie Y., Sun B., Guo Y., Song L., Mohammednur D.E., Zhao C. (2021). The Degradation of Rap1GAP via E6AP-Mediated Ubiquitin-Proteasome Pathway Is Associated with HPV16/18-Infection in Cervical Cancer Cells. Infect. Agents Cancer.

[31] Jiang H., Lei R., Ding S.-W., Zhu S. (2014). Skewer: A Fast and Accurate Adapter Trimmer for next-Generation Sequencing Paired-End Reads. BMC Bioinform..

[32] Dobin A., Davis C.A., Schlesinger F., Drenkow J., Zaleski C., Jha S., Batut P., Chaisson M., Gingeras T.R. (2013). STAR: Ultrafast Universal RNA-Seq Aligner. Bioinformatics.

[33] Love M.I., Huber W., Anders S. (2014). Moderated Estimation of Fold Change and Dispersion for RNA-Seq Data with DESeq2. Genome Biol..

[34] Liberzon A., Birger C., Thorvaldsdóttir H., Ghandi M., Mesirov J.P., Tamayo P. (2015). The Molecular Signatures Database Hallmark Gene Set Collection. Cell Syst..

[35] Subramanian A., Tamayo P., Mootha V.K., Mukherjee S., Ebert B.L., Gillette M.A., Paulovich A., Pomeroy S.L., Golub T.R., Lander E.S. (2005). Gene Set Enrichment Analysis: A Knowledge-Based Approach for Interpreting Genome-Wide Expression Profiles. Proc. Natl. Acad. Sci. USA.

[36] Kuleshov M.V., Jones M.R., Rouillard A.D., Fernandez N.F., Duan Q., Wang Z., Koplev S., Jenkins S.L., Jagodnik K.M., Lachmann A. (2016). Enrichr: A Comprehensive Gene Set Enrichment Analysis Web Server 2016 Update. Nucleic Acids Res..

[37] Chen E.Y., Tan C.M., Kou Y., Duan Q., Wang Z., Meirelles G.V., Clark N.R., Ma’ayan A. (2013). Enrichr: Interactive and Collaborative HTML5 Gene List Enrichment Analysis Tool. BMC Bioinform..

[38] Xie Z., Bailey A., Kuleshov M.V., Clarke D.J.B., Evangelista J.E., Jenkins S.L., Lachmann A., Wojciechowicz M.L., Kropiwnicki E., Jagodnik K.M. (2021). Gene Set Knowledge Discovery with Enrichr. Curr. Protoc..

