# Supplementary material for: Ficus carica Latex Modulates Immunity-Linked Gene Expression in Human Papillomavirus Positive Cervical Cancer Cell Lines: Evidence from RNA Seq Transcriptome Analysis
Source: Int J Mol Sci. 2023 Sep 4;24(17):13646. doi: 10.3390/ijms241713646 (PMC10488141; doi:10.3390/ijms241713646)
Supplement: Supplementary file 1 [file ijms-24-13646-s001.zip › ijms-2533047-supplementary.pdf]

ENSEMBL ID	Gene Name	Expression Status	Fold change (log_2 ratio)
ENSG00000006576	TSHZ2	Upregulated	4.605
ENSG00000010270	GLT8D2	Upregulated	5.841
ENSG00000020129	RASIP1	Upregulated	2.618
ENSG00000034510	NDRG1	Upregulated	4.255
ENSG00000040199	STOM	Upregulated	6.212
ENSG00000047634	PLEKHG4	Upregulated	5.362
ENSG00000048544	KLF4	Upregulated	5.33
ENSG00000053770	RMRP	Upregulated	6.467
ENSG00000056586	FBXO4	Upregulated	5.549
ENSG00000060642	GPCPD1	Upregulated	7.987
ENSG00000066933	HIST1H1E	Upregulated	5.732
ENSG00000070366	CTSF	Upregulated	3.788
ENSG00000076685	BET1	Upregulated	7.918
ENSG00000077514	TMEM14C	Upregulated	4.12
ENSG00000079335	BRIX1	Upregulated	7.706
ENSG00000081913	HIF1A	Upregulated	6.226
ENSG00000083457	TMEM126A	Upregulated	5.406
ENSG00000085491	CEP72	Upregulated	6.997
ENSG00000091157	CALR	Upregulated	7.985
ENSG00000099910	MZT1	Upregulated	5.134
ENSG00000100243	RAD1	Upregulated	3.068
ENSG00000100304	RPL27	Upregulated	7.426
ENSG00000100410	SUB1	Upregulated	7.192
ENSG00000100644	PRRT3.AS1	Upregulated	3.534

ENSG00000100888	PDCD6	Upregulated	7.213
ENSG00000101191	TMEM126B	Upregulated	4.164
ENSG00000104419	NELFE	Upregulated	4.619
ENSG00000105223	RHEBL1	Upregulated	7.161
ENSG00000105323	PLD3	Upregulated	6.522
ENSG00000105518	MRPS30	Upregulated	4.743
ENSG00000105538	MRPS10	Upregulated	4.161
ENSG00000105829	NFIL3	Upregulated	4.839
ENSG00000108100	CENPK	Upregulated	4.628
ENSG00000111696	DDX59	Upregulated	6.883
ENSG00000111843	C6orf136	Upregulated	5.69
ENSG00000112234	KLF13	Upregulated	5.772
ENSG00000112877	TMEM256	Upregulated	2.66
ENSG00000112996	CUL5	Upregulated	4.449
ENSG00000113387	SNRPD2	Upregulated	5.395
ENSG00000113456	RPS27A	Upregulated	4.309
ENSG00000113460	TMSB10	Upregulated	5.669
ENSG00000113761	DNAJC21	Upregulated	7.386
ENSG00000115942	GAS5	Upregulated	5.269
ENSG00000115944	PAIP1	Upregulated	5.528
ENSG00000118058	RPL37	Upregulated	5.679
ENSG00000118197	CHMP1B	Upregulated	5.898
ENSG00000119004	MRPS23	Upregulated	4.903
ENSG00000120820	RNF111	Upregulated	4.983
ENSG00000122786	GPR108	Upregulated	5.103

ENSG00000123219	COA4	Upregulated	4.777
ENSG00000123562	CYB5R3	Upregulated	4.75
ENSG00000123636	MRPL14	Upregulated	3.646
ENSG00000125734	SLC6A9	Upregulated	6.396
ENSG00000125743	MORF4L2	Upregulated	4.573
ENSG00000125772	TBC1D7	Upregulated	4.731
ENSG00000127337	ATP6AP2	Upregulated	6.933
ENSG00000127870	TTLL12	Upregulated	3.29
ENSG00000130511	POC1B	Upregulated	2.609
ENSG00000131469	RPL39	Upregulated	4.097
ENSG00000131725	GPR180	Upregulated	7.868
ENSG00000132646	SCML1	Upregulated	6.991
ENSG00000133789	HNRNPUL1	Upregulated	2.927
ENSG00000134308	MANEAL	Upregulated	7.291
ENSG00000135870	TMEM205	Upregulated	6.691
ENSG00000136098	ITGAE	Upregulated	5.203
ENSG00000136826	FAM105B	Upregulated	5.336

ENSG00000136943	WDR44	Upregulated	4.242
ENSG00000137154	PTMA	Upregulated	6.087
ENSG00000139323	SSBP4	Upregulated	4.969
ENSG00000139436	PRIM1	Upregulated	7.513
ENSG00000140199	POLD3	Upregulated	4.764
ENSG00000143947	PCNA	Upregulated	3.238
ENSG00000144426	FBXL4	Upregulated	5.398
ENSG00000145592	YEATS4	Upregulated	4.693
ENSG00000145979	WBSCR22	Upregulated	5.846
ENSG00000147654	C19orf66	Upregulated	5.101
ENSG00000148175	NEK3	Upregulated	4.88
ENSG00000148296	ORC2	Upregulated	3.568
ENSG00000151461	RPS6	Upregulated	3.233
ENSG00000151876	YIPF6	Upregulated	4.775
ENSG00000152102	LINC00910	Upregulated	5.103
ENSG00000152520	COX7A2L	Upregulated	5.97
ENSG00000152749	C2orf47	Upregulated	5.75

ENSG00000153827	EBAG9	Upregulated	5.357
ENSG00000157450	YWHAQ	Upregulated	4.579
ENSG00000158321	NBEAL1	Downregulated	-4.419
ENSG00000163249	CCDC137	Downregulated	-6.217
ENSG00000165030	NCDN	Downregulated	-5.203
ENSG00000166233	ZNF346	Downregulated	-2.771
ENSG00000166266	RNF115	Downregulated	-5.386
ENSG00000166987	ARIH1	Downregulated	-4.746
ENSG00000167550	STARD3NL	Downregulated	-4.598
ENSG00000168014	DDRGK1	Downregulated	-3.885
ENSG00000168298	SLC25A24	Downregulated	-3.257
ENSG00000168724	PHF5A	Downregulated	-7.23
ENSG00000169217	UPF2	Downregulated	-4.83
ENSG00000169925	CCNY	Downregulated	-2.36
ENSG00000169926	GIT2	Downregulated	-3.702
ENSG00000171202	CALD1	Downregulated	-6.634
ENSG00000171204	BRD3	Downregulated	-2.495

ENSG00000172239	C2CD3	Downregulated	-4.514
ENSG00000173575	PHTF2	Downregulated	-2.961
ENSG00000174080	AP5M1	Downregulated	-6.197
ENSG00000176371	SLC35E2	Downregulated	-2.481
ENSG00000176834	PAN3	Downregulated	-6.989
ENSG00000177000	ZNF652	Downregulated	-3.871
ENSG00000179218	CHD8	Downregulated	-5.72
ENSG00000180992	SURF6	Downregulated	-2.464
ENSG00000181610	TRIP12	Downregulated	-4.376
ENSG00000181704	CTSV	Downregulated	-2.713
ENSG00000181924	RC3H2	Downregulated	-4.831
ENSG00000182220	MBD6	Downregulated	-5.47
ENSG00000182463	PIP4K2B	Downregulated	-2.262
ENSG00000183723	DIDO1	Downregulated	-6.276
ENSG00000185090	NT5C2	Downregulated	-3.334
ENSG00000185252	RNF6	Downregulated	-7.111
ENSG00000185298	PIGV	Downregulated	-3.012

ENSG00000187514	CD2BP2	Downregulated	-6.644
ENSG00000188825	RP11.427H3.3	Downregulated	-6.393
ENSG00000196155	RP11.697E2.7	Downregulated	-4.879
ENSG00000196517	WDR7	Downregulated	-5.236
ENSG00000198056	CCNYL1	Downregulated	-3.639
ENSG00000198171	MYO9A	Downregulated	-2.981
ENSG00000198538	SLC12A6	Downregulated	-6.905
ENSG00000198740	ZSCAN2	Downregulated	-4.985
ENSG00000198918	RC3H1	Downregulated	-5.259
ENSG00000204356	CMTM4	Downregulated	-5.374
ENSG00000204564	SWAP70	Downregulated	-7.211
ENSG00000204899	DNASE1	Downregulated	-3.306
ENSG00000205544	CDC14A	Downregulated	-4.274
ENSG00000213918	ZNF74	Downregulated	-5.024
ENSG00000215421	FAM168B	Downregulated	-5.109
ENSG00000226266	NT5DC3	Downregulated	-4.581
ENSG00000234741	CYP20A1	Downregulated	-3.658

ENSG00000249915	ZNF28	Downregulated	-3.392
ENSG00000255112	CHD2	Downregulated	-6.552
ENSG00000265491	ZNF407	Downregulated	-5.567
ENSG00000276293	KMT2A	Downregulated	-5.327
ENSG00000277027	SMG6	Downregulated	-5.69
ENSG00000123636	BAZ2B	Downregulated	-4.941
ENSG00000081913	PHLPP1	Downregulated	-5.391
ENSG00000235499	AC073046.25	Downregulated	-5.763
ENSG00000263271	RP11.1055B8.7	Downregulated	-3.152
ENSG00000176834	VSIG10	Downregulated	-6.344
ENSG00000250899	RP11.253E3.3	Downregulated	-5.319
ENSG00000176834	MTHFR	Downregulated	-4.645
ENSG00000099910	KLHL22	Downregulated	-4.285
ENSG00000040199	PHLPP2	Downregulated	-4.35
ENSG00000158321	AUTS2	Downregulated	-6.557
